# Redox‐Active Polyphenol Red Molecularly Imprinted Polymers on Porous Gold Electrodes for Ultrasensitive, AI‐Assisted Detection of Alzheimer's Biomarkers in Undiluted Biofluids

**DOI:** 10.1002/adhm.202503155

**Published:** 2025-09-04

**Authors:** Sudhaunsh Deshpande, Ajith Mohan Arjun, Guoyi Liu, Kryztof Pawlak, Sanjiv Sharma

**Affiliations:** ^1^ David Price Evans Global Health and Infectious Diseases Group Pharmacology & Therapeutics Institute of Systems Molecular and Integrative Biology University of Liverpool Crown Street Liverpool L69 7BE UK; ^2^ Key Laboratory of Optoelectronic Technology & Systems (Chongqing University) Chongqing 400044 China; ^3^ Materials Innovation Factory University of Liverpool 51 Oxford Street Liverpool L7 3NY UK

**Keywords:** Alzheimer's Disease, point of care diagnostics, electrochemical biosensors, pTau 181, polyphenol Red

## Abstract

Early diagnosis of Alzheimer's disease (AD) is hindered by the high cost, complexity, and centralization of current diagnostic platforms such as enzyme‐linked immunosorbent assay (ELISA) and single‐molecule array (SIMOA). Here, an integrated point‐of‐care (PoC) biosensing platform is reported based on redox‐active polyphenol red molecularly imprinted polymers (pPhR MIPs) deposited on highly porous gold (HPG) electrodes for the ultrasensitive, reagent‐free detection of phosphorylated tau 181 (p‐tau 181) in undiluted plasma and serum. The unique electrochemical interface combines the signal‐enhancing properties of HPG with the redox functionality of pPhR, eliminating the need for external redox probes. The resulting sensor achieves a limit of detection (LOD) of 980 fg mL^−1^ and sensitivity of 4.39 µA/(pg mL^−1^), rivalling high‐end lab‐based assays while operating at a fraction of the cost. The platform integrates a low‐cost, handheld potentiostat and a machine learning (ML)‐enabled web app for real‐time data classification and regression, achieving 100% classification accuracy with zero false positives or negatives. The system demonstrates compatibility with both ethylenediaminetetraacetic acid (EDTA) and heparin‐treated samples and maintains accuracy in complex matrices. This work represents a step toward World Health Organization (WHO)‐compliant, decentralized diagnostics for neurodegenerative disease, offering a scalable foundation for future multianalyte platforms targeting AD and related disorders.

## Graphical abstract

This work presents a low‐cost, reagent‐free point‐of‐care biosensor integrating redox‐active polyphenol red molecularly imprinted polymers (pPhR MIPs) on highly porous gold (HPG) electrodes for ultrasensitive detection of phosphorylated tau 181 (p‐Tau 181), an Alzheimer’s disease biomarker, in undiluted biofluids. The platform incorporates a custom potentiostat and artificial intelligence‐assisted data analysis, enabling rapid, accurate, and portable neurodegenerative diagnostics.

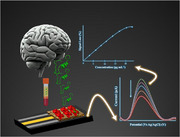

## 1 Introduction

The escalating global prevalence of neurodegenerative disorders—particularly Alzheimer's disease (AD), the leading cause of dementia—presents a mounting challenge to healthcare systems. Characterized by progressive memory loss, cognitive decline, and ultimately the loss of independent function, AD exerts a profound socioeconomic burden. Early onset symptoms may appear as early as the mid‐30s, while late onset typically begins after age 60.^[^ ^1^ ^]^ In 2017, over 121000 deaths were linked to AD in the United States alone. The caregiving burden, valued at over US$234 billion in unpaid care, reflects the enormous physical, emotional, and financial toll.^[^ ^2^ ^]^ The situation is projected to intensify, with healthcare and hospice costs for patients aged 65 and above surpassing US$290 billion in 2019, alongside a 145% increase in cases between 2000 and 2017.^[^ ^2^ ^]^

Although AD progression is irreversible, early diagnosis can facilitate timely intervention, improving patient outcomes through medical management, lifestyle modifications, and access to clinical trials.^[^ ^3^ ^]^ Existing diagnostic strategies rely on imaging techniques—including MRI, fMRI, sMRI, and PET scans—to assess structural and functional brain changes and identify key biomarkers such as amyloid β (Aβ) and tau proteins.^[^ ^3^, ^4^ ^]^ Alternatively, fluid‐based diagnostics analyse cerebrospinal fluid (CSF) for biomarkers including Aβ42/40, neurofilament light (NfL), and phosphorylated tau (p‐tau 181).^[^ ^5^ ^]^ While enzyme‐linked immunoassays (ELISA) and single‐molecule array (SIMOA) platforms enable high‐sensitivity detection in CSF and plasma,^[^ ^5^, ^6^ ^]^ they are hindered by the need for sophisticated infrastructure, skilled operators, and sample pre‐processing, limiting accessibility in decentralized or resource‐constrained settings.

To address these gaps, there is growing interest in point‐of‐care (PoC) diagnostics capable of delivering rapid, low‐cost, and minimally invasive testing. Among accessible sample types, plasma and serum are highly attractive, but their complex biological matrices pose significant challenges—particularly biofouling, caused by nonspecific protein adsorption that interferes with signal interpretation. Common antifouling strategies employ surface coatings like PLA, TMOS sol‐gels, or PLL‐PEG, but these often attenuate signal strength.^[^ ^7^ ^]^ Commercial solutions such as STATA DX's BSA/AuNW/GA nanocomposites show promise,^[^ ^8^ ^]^ but introduce cost and fabrication complexity. In contrast, highly porous gold (HPG) offers an elegant solution, providing hydrophobic, antifouling surfaces while significantly amplifying electrochemical signals due to increased electrochemically active surface area (ECSA).^[^ ^9^, ^10^, ^11^ ^]^

Modifying a surface with HPG increases the electrochemically active surface area (ECSA) of the sensing surface, which results in signal amplification.^[^ ^10^ ^]^ Central to any biosensor is its biorecognition element. Antibodies—commonly used for p‐tau 181 detection—offer excellent specificity but suffer from cost, instability, and reliance on redox probes,^[^ ^12^, ^13^, ^14^ ^]^ Aptamers, while tuneable and highly specific,^[^ ^15^ ^]^ require the labour‐intensive SELEX process, making them impractical for scalable or low‐resource deployment. Molecularly imprinted polymers (MIPs) offer a robust alternative: synthetic, low‐cost, and highly stable materials capable of mimicking natural antibody binding. MIPs are tailored to recognize target molecules via complementary cavities formed during polymerization,^[^ ^16^, ^17^ ^]^ However, traditional MIPs may suffer from non‐specific binding, which can be mitigated through surface modification and antifouling substrates like HPG.^[^ ^18^ ^]^

Despite these advances, many MIP‐based sensors still rely on external redox probes (e.g., Fe^2^⁺/Fe^3^⁺) to transduce binding events. To eliminate this dependency, our group has explored in situ redox‐active systems, including PDA‐based MIPs with MXene substrates for tau 441 detection.^[^ ^19^, ^20^ ^]^ However, such platforms are limited by MXene's poor stability in oxidative environments and the coffee ring effect from drop‐casting, which hampers reproducibility. We address these issues by employing polyphenol red (PhR)—a redox‐active dye polymer that, when electropolymerized, forms a stable layer with low oxidation potential (≈0.08 V), reducing interference from redox‐active bioanalyses such as ascorbic acid and paracetamol.^[^ ^21^, ^22^ ^]^ PhR's sulfonate and hydroxyl groups also enable hydrogen bonding and electrostatic interactions with phosphate groups on p‐tau 181, providing molecular specificity.

In parallel, we replace costly screen‐printed electrodes (SPEs)—typically priced at $8) per unit—with custom‐designed printed circuit boards (PCBs). PCBs are scalable, cost as little as £0.09 per unit, and support multiplexing, but require surface modification to overcome the electrochemical inactivity of their standard electroless nickel immersion gold (ENIG)finish. We resolve this by introducing a silver barrier layer and electroplated gold, enabling stable electrochemical performance. Additionally, we developed a low‐cost, portable potentiostat to interface with the sensor, eliminating the need for benchtop instrumentation. By integrating machine learning (ML) into the system, we enable automated signal classification and regression analysis, mitigating user bias and enhancing diagnostic reliability. To further refine the sensor's analytical capabilities and translate complex electrochemical signals into clear, quantitative results, an ML approach was integrated into the system. By employing advanced algorithms, it is possible to model the non‐linear dose‐response behavior, correct for minor variations between sensors, and enhance the overall accuracy and reliability of measurements (**Table** **1**).^[^ ^23^, ^24^, ^25^, ^26^, ^27^ ^]^

**Table 1:** Shows some of the current technologies developed for the detection of tau proteins.

Title	Bioreceptor	Nanomaterial	In situ redox probe	Dynamic range	Limit of detection	Sample matrix	Reference
Development of a biosensor for phosphorylated Tau 181 protein detection in Early‐Stage Alzheimer's disease	Anti‐p‐tau181 Antibody	Multi‐Walled Carbon Nanotubes (MWCNTs) and Platinum Nanoparticles (PtNPs)	No	8.6–1100 pg mL^−1^	0.24 pg mL^−1^	Human serum	[12]
Novel Electrochemical Molecularly Imprinted Polymer‐Based Biosensor for Tau Protein Detection	3‐aminophenol MIP (Tau 441)	N/A	No	0.14824–148.24 ng mL^−1^ (in PBS)	1.36 pg mL^−1^ (in PBS)	Human serum	[28]
Fully Integrated Wearable Biosensor for Multiple In Situ Phosphorylated Tau Protein Detection	Anti‐p‐tau181 Antibody	N/A	No	0.1–1000 pg mL^−1^	0.058 pg mL^−1^	Mouse ISF	[29]
Novel biomimetic Prussian blue nanocubes‐based biosensor for Tau‐441 protein detection	3‐aminophenol MIP (Tau 441)	Graphene Oxide/Prussian Blue Nanocubes (GO/PBNCs)	Prussian Blue Nanocubes	74.12–148.24 ng mL^−1^	0.680 pg mL^−1^	PBS	[30]
Alzheimer's diagnosis beyond cerebrospinal fluid: Probe‐Free Detection of Tau Proteins using MXene based redox systems and molecularly imprinted polymers	Poly‐aniline, poly‐dopamine MIP (Tau 441)	Vanadium MXene (V_x_)	Poly‐dopamine	74.12–148.24 pg mL^−1^	0.680 pg mL^−1^	Artificial ISF and serum	[19]
Machine Learning‐Assisted Sensor Array Based on Poly(amidoamine) (PAMAM) Dendrimers for Diagnosing Alzheimer's Disease	Poly(amidoamine) (PAMAM) Dendrimers (tau)	N/A	N/A	0.1–100 ng mL^−1^	3.38 fg mL^−1^	Human serum	[23]
Electrochemical Tau Protein Immunosensor Based on MnS/GO/PANI and Magnetite‐incorporated Gold Nanoparticles	Anti‐Tau Antibody	MnS/GO/PANI and AuNPs@Fe3O4	No	6.8 pg mL^−1^ to 68 µg mL^−1^	0.68 pg mL^−1^	PBS	[31]
High‐Performance Plasma Biomarker Panel for Alzheimer's Disease Screening Using a Femtomolar‐Level Label‐Free Biosensing System	Anti‐p‐Tau 181 Antibody	Graphene‐based Field‐Effect Transistor (gFET)	N/A	1 fg mL^−1^ to 1 ng mL^−1^	Not specified	Human plasma	[32]
Electrochemical impedance‐based biosensor for label‐free determination of plasma P‐tau181 levels for clinically accurate diagnosis of mild cognitive impairment and Alzheimer's disease	Anti‐p‐Tau 181 Antibody	Reduced Graphene Oxide/β‐cyclodextrin (rGO/β‐CD) nanocomposite	No	10⁻^3^ to 10^3^ pg mL^−1^	0.92 fg mL^−1^	Human plasma	[13]
Vertical Graphene‐Based Printed Electrochemical Biosensor for Simultaneous Detection of Four Alzheimer's Disease Blood Biomarkers	Anti‐p‐Tau 181 Antibody	Vertical Graphene (VG) modified with Gold Nanoparticles (AuNPs).	No	0.1 pg mL^−1^ to 1 ng mL^−1^	0.051 pg mL^−1^	1:3 dilute human serum	[33]
This work	Phenol red MIP (p‐Tau 181)	Highly porous gold (HPG)	Phenol red	0.5–22.5 pg mL^−1^	0.98 pg mL^−1^	PBS, undiluted human serum and plasma	

**Figure 1:**
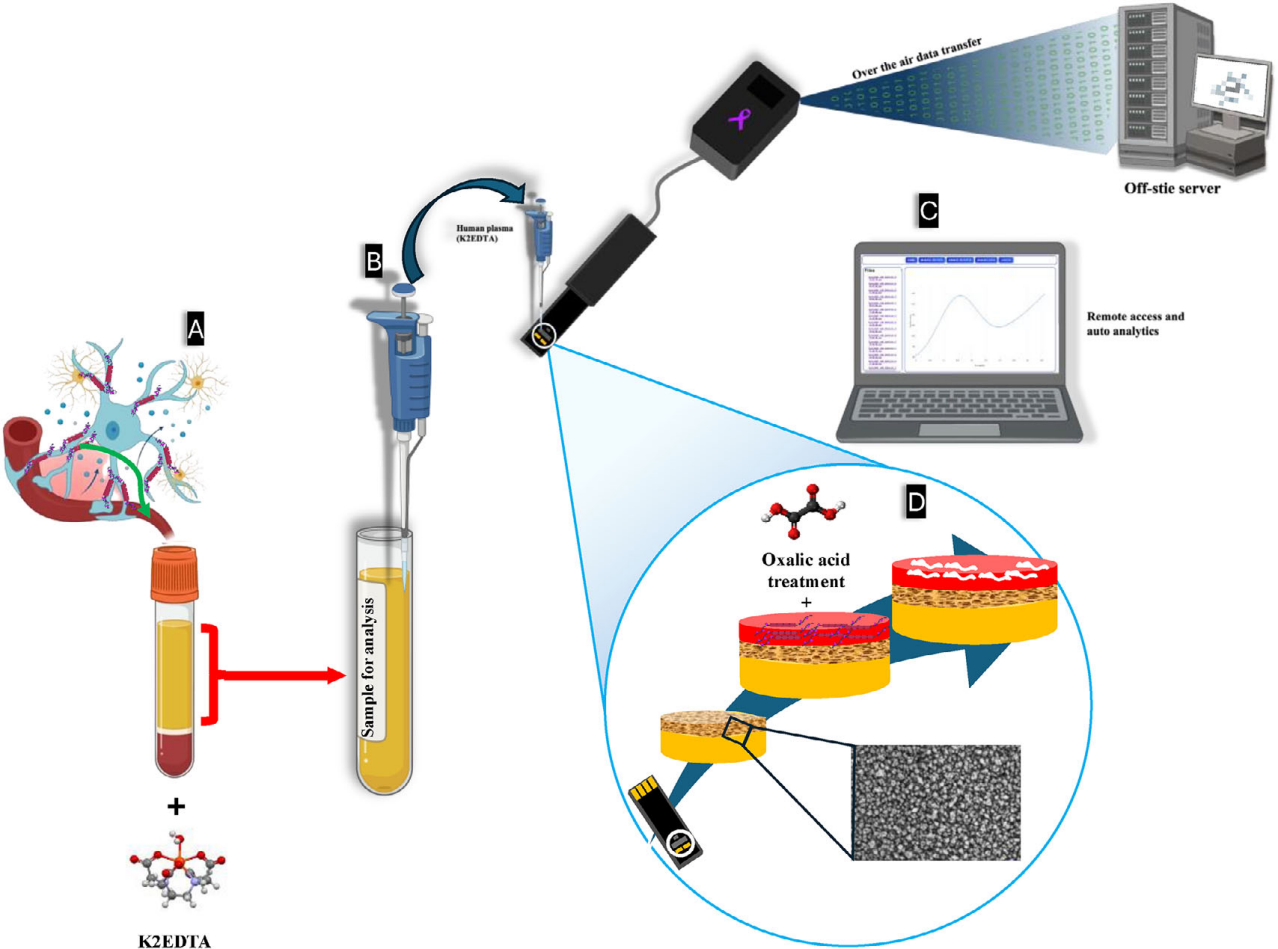
An overview of the biosensing system presented in this work. A) Illustration of the p‐tau 181 protein crossing the blood‐brain barrier, and collection of blood in a tube with EDTA. B) Illustrating the separation of plasma and dropping plasma on the biosensor. C) Illustration of the automated data analysis system. D) Illustration of the fabrication process of the biosensor, with an inset of a SEM image of the highly porous gold surface.

Here, we present a scalable, AI‐integrated PoC biosensor leveraging redox‐active polyphenol red MIPs and HPG electrodes for direct detection of p‐tau 181 in undiluted plasma and serum (**Figure** 1). This platform demonstrates ultrasensitive detection (LOD: 980 fg mL^−1^; sensitivity: 4.39 µA pg^−1^ mL^−1^), robust performance in anticoagulated samples, and 100% classification accuracy via supervised ML. With its low cost, reagent‐free operation, and compatibility with complex matrices, this work lays the foundation for decentralized AD diagnostics, in alignment with the guidelines issued by the World Health Organization (WHO) for accessible and equitable testing.^[^ ^34^ ^]^

## 2 Experimental Section

### 2.1 Reagents and Materials

Silver and gold brush plating solutions were procured from Spa Plating (Bath, UK). Gold (III) chloride (AuCl_3_), ammonium chloride (NH_4_Cl), phenol red sodium salt (chosen for its redox activity and compatibility with polymerization techniques), ferric chloride, and oxalic acid were sourced from Sigma‐Aldrich (St. Louis, MO, USA). Recombinant p‐tau 181 protein was purchased from Abcam (Cambridge, UK). Printed circuit boards (PCBs) with ENIG surface finishes were obtained from JLC PCB (Shenzhen, China). Human serum was procured from Sigma‐Aldrich (Merck) (**H4522** from human male AB plasma, USA origin). Human plasma was procured from Cambridge Biosciences (UK).

### 2.2 Electrode Preparation

**Figure 2:**
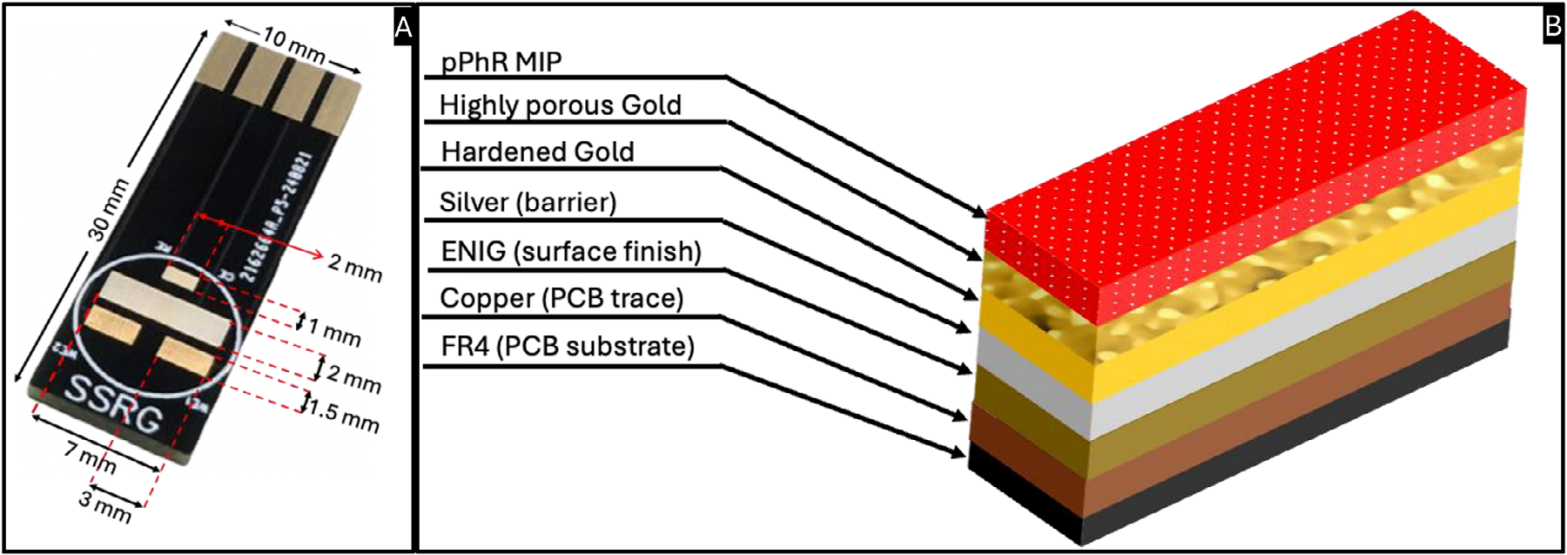
An overview of the bioinstrumentation. A) Image of a surface‐modified PCB (Bare Au) with dimensions of the electrodes. B) Illustration of the layers of the functionalized surface.

**Figure 3:**
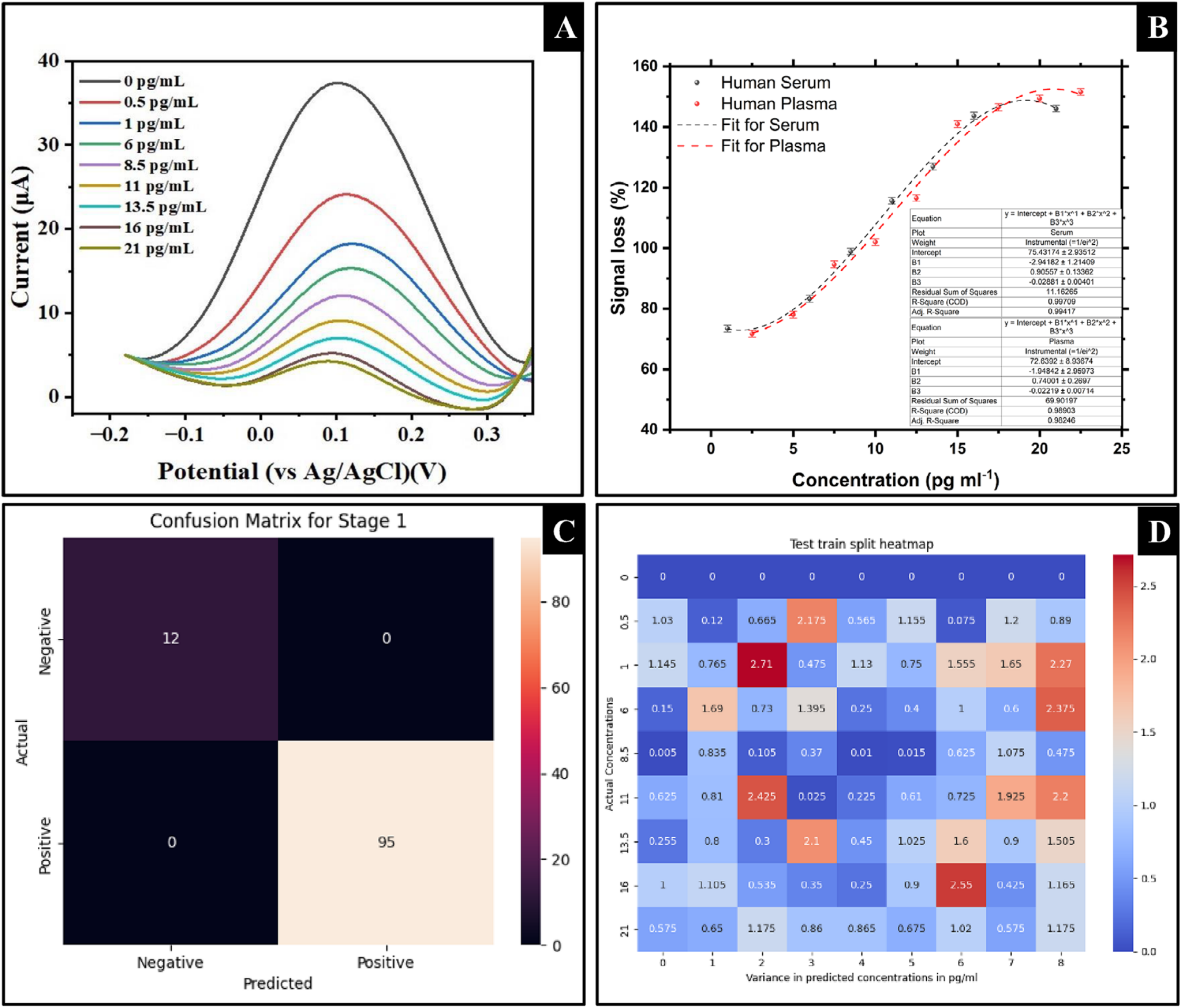
Sensor Performance: A) Differential Pulsed Voltammograms in p‐Tau 181 spiked human serum samples. B) Dose response in human serum and (K2EDTA) human plasma. C) Confusion matrix output used to cross‐validate the classifier using the scikit metrics and test‐train split libraries. D) Heatmap displaying in variance of data versus expected values for the regressor using the scikit metrics and test train split libraries.

The PCB design consist of four islands two 3 mm × 1.5 mm (to be used as Working Electrode (WE) 1 and WE2), one 7 mm × 2 mm island, to be used as the counter electrode (CE) and one 2 mm × 1 mm island to be used as the reference electrode (RE) (**Figure** **2**A). A silver layer was deposited on all electrodes using a silver brush plating solution. Silver acts as a barrier layer to the ENIG surface, as the Nickel in this composite is electrochemically active and renders the surface unsuitable for performing electrochemical measurements (**Figure** **3**A).^[^ ^35^ ^]^ The silver deposition was carried out using a potentiostatic cell setup with the Potentiostat (Palmsense Emstat 4) WE connected to the PCB islands, RE connected to a CH Instruments standard glass junction Ag/AgCl reference electrode, and the CE was connected to a Pt/Ti type electrode (spa plating). Multistep amperometry was performed at −0.5 and −1.2 V in 2.5‐s pulses for 16 cycles with stirring set to 300 rpm.

Gold electroplating was conducted on the working islands using a gold brush plating solution (Spa Plating) by running chronopotentiometry at −1.5 mA for 300 s with stirring at 300 rpm in a galvanic cell with Pt/Ti type CE. The RE island was chloridized by immersion in 100 mm ferric chloride solution for 30 s to form a silver chloride layer.

### 2.3 Highly Porous Gold Surface Modification

The modification of the PCB/ Au with HPG was carried out according to a procedure reported by Bollela et al.^[^ ^9^ ^]^ A potentiostatic cell was set up wherein the PCB WE island of the PCB electrode was connected to the Potentiostat WE, a Pt electrode from ALS was used as the CE, and a CH Instruments standard glass junction Ag/AgCl electrode was used as the RE. Soft gold was deposited by the reduction of AuCl_3_. This was achieved by running Cyclic Voltammetry (CV) between 0.8 and 0.3 V at a scan rate of 0.05 V s^−1^ for 10 cycles in a freshly prepared solution of 10 mm AuCl_3_ and 2.5 M NH_4_Cl mixed in a 1:1 ratio. Electroporation was done by running Chronoamperometry (CA) at −0.9 V for 60 s.

### 2.4 Molecularly Imprinted Polymer Synthesis

A 5 mm Phenol red sodium salt solution was made in 10 mm phosphate buffered saline (PBS) and degassed under nitrogen for 15 min. The 5 µL of 0.2 mg mL^−1^ p‐tau 181 recombinant protein was mixed with 200 µL phenol red solution and 795 µL of PBS to achieve a final concentration of 1 µg mL^−1^. This solution, containing the phenol red monomer and the p‐tau 181 protein template, was used for electrochemical polymerisation.

Electrochemical polymerisation was conducted using the PCB electrodes (WE1 and WE2) as working electrodes, an ALS standard platinum disc CE, and the internal RE in a potentiostatic cell. The polymerization technique reported by W. Limbut was adopted and further optimized for this application.^[^ ^36^ ^]^ CV was performed from 0.3 to 0.8 V at a scan rate of 0.1 V s^−1^ for 18 cycles. The electrodes were rinsed in DI water and dried under a nitrogen stream. The electrodes were incubated overnight in 50 mm oxalic acid in a refrigerator to remove the template molecule, leaving cavities specific to p‐tau 181.

### 2.5 Bioinstrumentation

#### 2.5.1 Wireless Handheld Potentiostat

A wireless handheld potentiostat was developed using an ESP32‐D0WD‐V3 microcontroller. This microcontroller was chosen for its high‐resolution (12‐bit) analogue to digital converter (ADC), and most importantly, the dual‐channel onboard digital analogue converter (DAC).^[^ ^37^ ^]^ The DAC is used to control the driving potential of the CE, and the ADC is used to quantify the output potential from the potentiostat. The analogue front end (AFE) was designed around the LM324N quad‐channel (A,B,C, and D) operational amplifier (OP‐Amp).^[^ ^38^ ^]^ Op‐Amp A was set in a differential amplifier (aka comparator) configuration to drive the CE, whereas Op‐Amp B was configured as a buffer Op‐Amp feeding the buffer potential from the RE to the differential Op‐Amp A. The Op‐Amp C was configured to function as a transimpedance amplifier (TIA) to measure current from the Working Electrode. Finally, the Op‐Amp D was used in a comparator configuration to act as a potential divider such that it only provides positive potentials to the ESP32's ADC (ADC can only read positive potentials) (Figure S1A, Supporting Information). The potentiostat design incorporates a DC‐DC isolator to provide a virtual ground and essentially double the OP‐Amp potential window from 0 to +5 V to −5 to +5 V (Figure S1F,G, Supporting Information). The high common‐mode rejection of the Op‐Amp also helps in eliminating environmental noise.^[^ ^38^ ^]^ The data collected by the microcontroller is then serialized as a JSON file, which is transmitted to the server via WiFi (Figure S1B, Supporting Information).

#### 2.5.2 Multiplexer

A multiplexer (MUX) was designed complementary to the Potentiostat using an LH1502BAC optocoupled solid state relay and an Arduino Nano, to switch between WE1 and WE2 of the sensor. The Arduino Nano and the ESP32 communicate using the I2C bus to automatically switch channels during measurements.

#### 2.5.3 Machine Learning Web App

A supervised machine learning (ML) model was designed using the DecisionTree and RandomForest classifiers and regressors from Sci‐kit. The classifiers and regressors work together for a comparative analysis and to reduce the false readouts. The features used to train the model were peak width (V (Vs Ag/AgCl)), peak potential (V (Vs Ag/AgCl)), peak height (µA), signal loss (%), and concentration (pg mL^−1^). The models were trained on a total of 4500 data points collected in human plasma. Cross‐validation was done by using the scikit test train split function by splitting the dataset into a training and test dataset. The resulting output data is shown in Figure 3C,D. The algorithm is integrated into a web app based on Flask, a Python library (Figure S1C, Supporting Information). The models were trained using automated feature extraction algorithms. These algorithms produce a (Pandas) dataset with feature columns. The server runs on Gunicorn, a Web Server Gateway Interface (WSGI). This system accepts the JSON file from the Potentiostat and deserializes the data, and saves it locally on the server's allocated storage. The data is then run through the feature extraction algorithm and the ML algorithm to generate the results (Figure S1D, Supporting Information). The data can be accessed by the user by accessing the web dashboard (Figure S1C, Supporting Information).

### 2.6 Electrochemical Characterization

Electrochemical characterization was performed using CV and differential pulse voltammetry (DPV) to assess the sensor's ECSA, redox behaviour, and binding specificity. A CV comparison between ENIG, bare planar gold, and HPG was done in a solution of 5 mm Fe^2+^ and Fe^3+^ ions with 100 mm KCl. The binding specificity of the sensor was compared by analysing the sensor performance in PBS, human serum, and human plasma, along with an assessment of a nonimprinted polymer in spiked plasma. Furthermore, the effect of anticoagulant was also studied by using human plasma with two anticoagulants ethylenediamine tetra acetic acid (EDTA) and heparin. A standard protocol was designed to measure the p‐tau 181 using this biosensor. The 70 µL of the sample was dropped on the sensor. The DPV was run from −0.2 to 0.5 V with an amplitude of 0.05 V, a potential step of 0.01 V at a scan rate of 0.1 V s^−1^. The sample was then allowed to incubate on the sensor surface for 30 min, followed by running another DPV with the same parameters to measure the signal loss.

### 2.7 Surface Characterization

Scanning Electron Microscopy (SEM) was conducted on various samples using a ZEIS Geminin 2. For achieving high‐resolution images at high vacuum settings and to reduce drift, the sensors were sputter‐coated with Chromium. During the SEM imaging process, Energy Dispersed X‐Ray (EDX) Spectroscopy was performed in the same machine.

Samples were analysed using a Renishaw Invia Raman microscope. The spectra were collected using 10% power of the equipped 532 nm laser at a 1 s exposure with a measurement window of 1570–350 cm^−1^. Raman maps were obtained with the StreamHR mode with a 10X objective. The instrument was calibrated using a silicon standard at 520.5 cm^−1^. The mapping area was set to 2700 µm × 1300 µm with 10 µm step. The Raman maps were obtained using Renishaw's Empty Modelling method to obtain the spatial distribution of the polymer.

Fourier Transform Infrared (FTIR) spectroscopy Attenuated Total Reflectance (ATR) was conducted on a Bruker Vertex 70 equipped with a Diamon MIRacle ATR accessory (Pike Technologies Spectroscopic Creativity) and a Deuterated L‐alanine doped Triglycine Sulphate (DLaTGS) detector. The size of the ATR crystal is 1.8 mm in diameter. The spectra were recorded in the 4000–400 cm^−1^ range with 4 cm^−1^ resolution. Each spectrum is a result of an average of 32 scans. All the data was processed using the Bruker OPUS 8.2 software.

## 3 Results and Discussion

### 3.1 Electrochemical Behaviour

The electrochemical behaviour of the sensing platform was first evaluated to assess the role of highly porous gold (HPG) in improving charge transfer. Cyclic voltammetry (CV) in Fe^2^⁺/Fe^3^⁺ solution revealed that HPG significantly enhanced the electrochemically active surface area (ECSA), producing a 2.42‐fold increase in current relative to planar gold (Figure 3A). A decrease in peak‐to‐peak separation (ΔEp) from 240 to 120 mV indicates faster electron transfer kinetics due to the nanostructured morphology.^[^ ^9^, ^10^, ^11^ ^]^

Following modification with electropolymerized polyphenol red (pPhR) MIPs, an oxidative peak at 0.08 V in 10 mm PBS was consistently observed (Figure 3B), validating the successful deposition of the redox‐active polymer. The low oxidation potential of pPhR offers a key advantage by minimizing interference from common serum interferents such as ascorbic acid and paracetamol.^[^ ^21^, ^22^ ^]^ This redox signal serves as an intrinsic transduction mechanism, eliminating the need for external redox probes. As target p‐tau 181 binds to MIP cavities, a proportional reduction in ECSA occurs, leading to a decrease in redox peak current—providing a label‐free, quantitative readout.

To further address potential cross‐reactivity, we conducted a competitive binding study using phosphorylated amyloid‐beta (p‐Aβ(1–42), ≈4.5 kDa), a structurally dissimilar but phosphate‐containing peptide present in AD serum. The results, presented in Supporting Figure S3 (Supporting Information) (Panels A–C), show minimal signal interference, highlighting the high molecular selectivity of our pPhR MIP sensor for p‐Tau181. These findings are further supported by our molecular docking and energy calculations, which demonstrate preferential binding affinity and steric compatibility between the MIP cavities and p‐Tau181.^[^ ^39^ ^]^ The selection of p‐Aβ_(1‐42)_ as the nontarget analyte is critical, as it not only co‐exists with p‐tau 181 in AD pathology but also shares the essential phosphoserine recognition motif. This presents a worst‐case scenario for potential cross‐reactivity, allowing for a stringent test of whether the MIP's selectivity is driven by the overall molecular template or merely by the presence of a phosphate group.

We employed molecular docking simulations to computationally probe the binding interactions of both p‐tau 181 (≈68 kDa) and p‐Aβ_(1‐42)_ within the imprinted MIP cavity. The results were unequivocal: the target p‐tau 181 peptide docked with high affinity (−4.38 ± 0.038 KJ mol^−1^) (Figure S3B, Supporting Information), demonstrating favourable binding energies and extensive shape complementarity. Conversely, the non‐target p‐Aβ_(1‐42)_ peptide failed to form a stable complex. Despite sharing the phosphorylated residue, its distinct size and 3D structure led to a profound steric mismatch with the imprinted cavity (7.92 ± 0.326 KJ mol^−1^) (Figure S3C, Supporting Information). These simulations support that the pPhR‐MIP operates on a principle of holistic structural recognition and structurally oriented interaction mechanisms, ensuring high selectivity for p‐tau 181 and effectively preventing misidentification of other similarly modified biomarkers.

### 3.2 Sensor Specificity, Sensitivity, and Clinical Compatibility

The antifouling performance of the HPG surface was evaluated using a **nonimprinted polymer (NIP)** in p‐tau 181–spiked plasma. The negligible signal variation across concentrations (Figure S2C, Supporting Information) confirms that **biofouling was effectively mitigated**, likely due to the **hydrophobic, nanostructured surface** of HPG.

Dose‐response curves in PBS, human serum, and plasma (Figure 3B) showed consistent performance across matrices, with a limit of detection (LOD) of 980 fg mL^−1^, upper limit of quantification (ULOQ) in plasma of 22.5 pg mL^−1^ (relative standard deviation (RSD = 13.8125%) and relative error (RE = −8%) ≤ 15% and ≤ ±15% respectively) and an average sensitivity of 4.23 µA/(pg mL^−1^). Notably, this sensor operates directly in undiluted biofluids, avoiding the preprocessing steps required by ELISA and SIMOA.^[^ ^40^ ^]^ The response remained robust in both EDTA‐ and heparin‐treated plasma (Figure S2E, Supporting Information), unlike commercial assays that require anticoagulant‐specific calibration. This demonstrates the platform's clinical adaptability, even in decentralized testing scenarios.

To contextualize performance, Table S1 (see Supporting Information) compares LOD, assay time, and other factors with SIMOA, ELISA, and MIP‐based sensors. This sensor outperforms other MIP platforms in both simplicity and sensitivity while approaching the performance of SIMOA at significantly lower cost.

### 3.3 AI‐Enhanced Biosensing and Predictive Analytics

To improve usability and reproducibility, the sensor was integrated with a machine learning–driven web app for real‐time classification and regression. A decision tree classifier trained on features such as peak current, signal loss, and sample ID yielded 100% classification accuracy, with zero false positives or negatives (Figure 3C). These results confirm the algorithm's robustness and the distinct electrochemical signatures produced upon p‐tau 181 binding.

A random forest regressor further quantified p‐tau 181 levels. The low variance between predicted and measured concentrations is visualized in the heatmap (Figure 3D). To strengthen validation, future iterations will replace this with a Bland–Altman plot to highlight agreement across the detection range. The most influential features in the model were signal loss magnitude and peak shift, which correlate directly with target binding events. This integration of supervised ML ensures automated, unbiased interpretation of electrochemical data, a key requirement for PoC diagnostics in resource‐limited settings.

### 3.4 Surface Morphology and Chemical Characterisation

**Figure 4:**
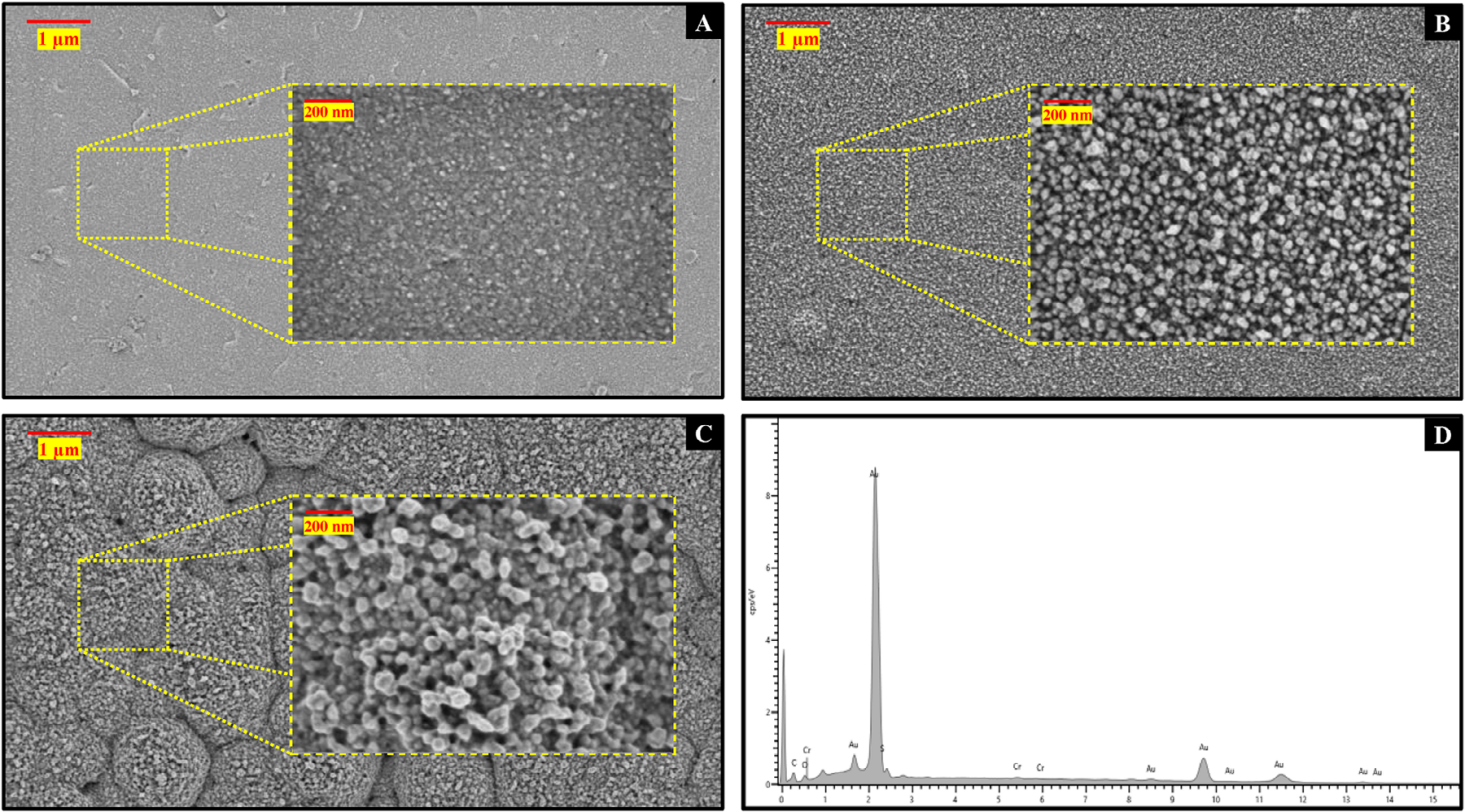
Surface characterisation and elemental analysis: A) SEM image of the bare gold electrode. B) SEM image of the highly porous gold modification. C) SEM image of the MIP sensing surface. D) Energy Dispersed X‐Ray spectrum of the sensing surface.

SEM analysis confirmed distinct morphological transitions at each fabrication step. The bare gold surface (**Figure** **4**A) exhibited smooth, uniform grains. Following electrochemical poration, HPG displayed a highly porous nanostructure (10–50 nm pores) (Figure 4B), dramatically increasing surface area (ECSA/geometric area: 45.22 cm^2^ cm^−^^2^). The ECSA was calculated using the Randles‐Sevick equation:

(1) $$i_{p}=0.4463\;nFAC\;\sqrt{\frac{nFvD}{RT}}$$

where i_p_ is the peak current of a redox reaction in a voltammogram (A), n is the number of electrons of the reaction, F is Farraday's constant (96 485 C mol^−1^), A is the ECSA (cm^2^), C is the concentration of redox probe (mol cm^−3^), v is the scan rate used to collect the voltammogram (V/s), D is the diffusion coefficient of the redox species (cm^2^ s^−1^), R is the gas constant (8.3144 J^−1^ mol K^−1^), and T is the temperature (K).^[^ ^41^ ^]^ This enhanced surface provides greater MIP loading and more binding sites, directly correlating with higher sensitivity and reduced biofouling.

After MIP deposition, a uniform polymer coating was observed (Figure 4C), suggesting successful and reproducible functionalization. EDX analysis (Figure 4D) confirmed the absence of silver and nickel, indicating complete coverage of the silver barrier layer and ENIG substrate. The presence of sulfur further validated the successful incorporation of pPhR's sulfonate groups.

### 3.5 Spectroscopic Validation of pPhR‐MIP Formation

**Figure 5:**
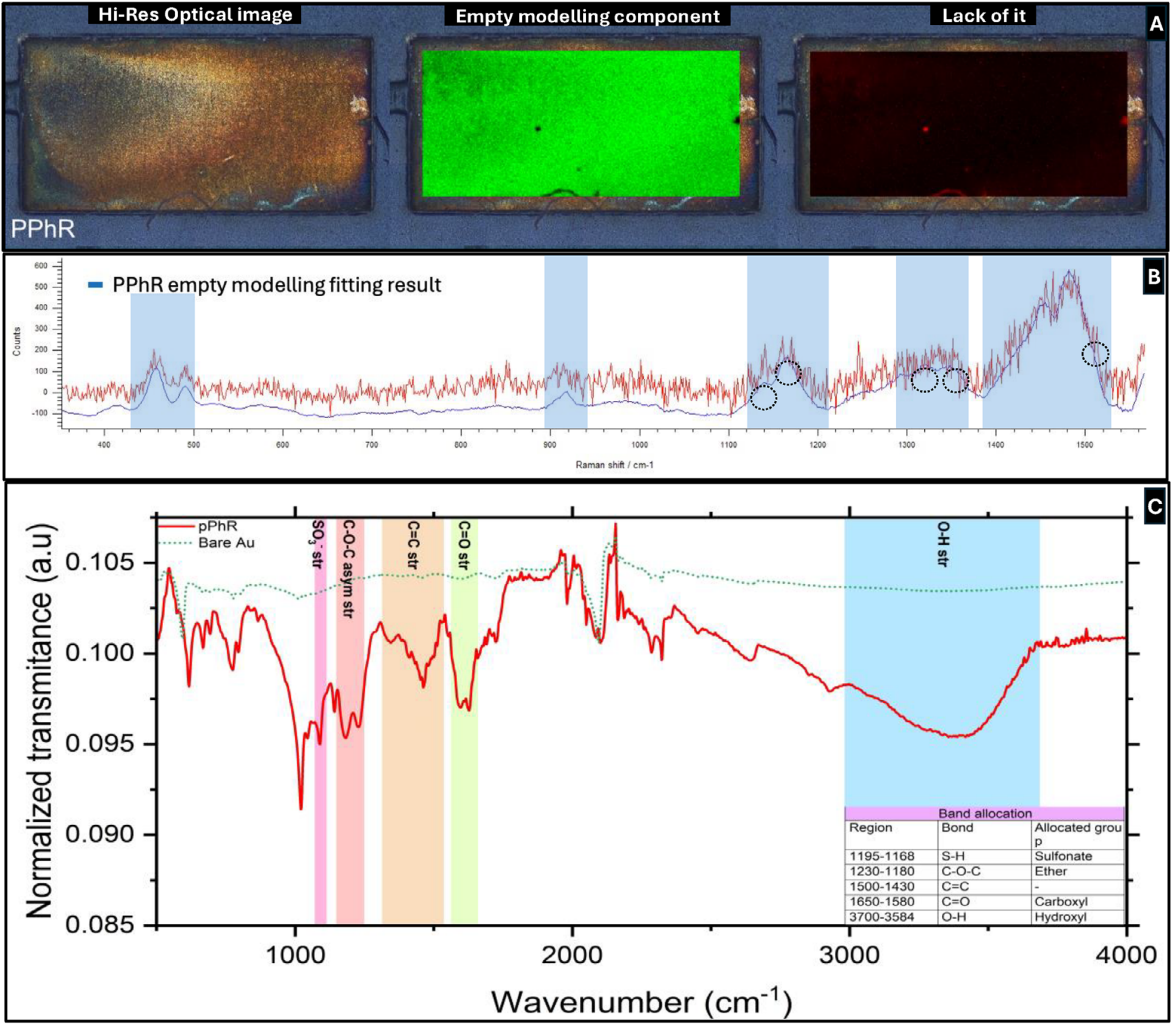
Chemical characterisation of the sensing surface. A) Image of Raman mapping, depicting the optical image and the uniformity of the polymer. B) Average Raman spectrum and empty model spectrum. C) FTIR ATR spectra comparing unmodified bare gold and MIP surfaces.

Raman spectroscopy identified characteristic pPhR peaks at 1127, 1183, 1310, 1362, and 1500 cm^−1^, corresponding to C─C bond vibrations and xanthene ring deformations, consistent with previous studies.^[^ ^42^ ^]^ Mapping via Empty Modelling revealed uniform polymer coverage, with minor discontinuities likely due to debris (**Figure** **5**A,B).

FTIR–ATR analysis (Figure 5C) confirmed sulfonate group peaks at 1043 cm^−1^, C─O─C stretching between 1125 and 1275 cm^−1^, and aromatic C═C stretching ≈1320–1550 cm^−1^. A broadened C═O peak ≈1600 cm^−1^ indicated successful polymerization of the quinone methide ring.^[^ ^43^, ^44^ ^]^ Together, these findings validate the structural integrity and chemical specificity of the MIP layer.

### 3.6 Comparison with Existing Diagnostic Methods

Compared to gold‐standard assays, this sensor provides competitive sensitivity at a fraction of the cost and complexity. For instance, SIMOA's p‐tau 181 kit reports a LOD of 620 fg mL^−1^, but requires specialist instrumentation, dilution steps, and high per‐test costs (∼£6000/plate).^[^ ^40^ ^]^ By contrast, our platform achieves comparable sensitivity (980 fg mL^−1^) using a handheld potentiostat, operates on raw samples, and is fabricated for under £20 per test. Unlike MXene‐ or PDA‐based MIPs, the pPhR‐MIP avoids redox probe addition and offers greater environmental and signal stability.^[^ ^19^, ^20^ ^]^

### 3.7 Clinical Implications and Future Outlook

This sensor enables quantitative detection of p‐tau 181 in minimally processed plasma and serum, supporting early‐stage Alzheimer's disease triaging in primary care or home settings. Beyond AD, the modular design allows extension to other neurodegenerative biomarkers (e.g., Aβ40/42, NfL, p‐tau 217), facilitating development of multiplexed biosensors for broader neurological diagnostics.

Ongoing efforts will focus on:

- Multiplexing sensor arrays on PCB for simultaneous multi‐biomarker detection
- Larger‐cohort clinical validation, particularly in early MCI cases
- Integration with mobile health platforms for real‐time, remote monitoring
- Alignment with WHO's equity‐focused diagnostics roadmap for neurodegeneration.^[^ ^45^ ^]^

## 4 Conclusion

We present a low‐cost, redox‐active molecularly imprinted polymer (MIP) biosensing platform based on polyphenol red (pPhR) integrated with highly porous gold (HPG) electrodes, enabling ultrasensitive, point‐of‐care (PoC) detection of phosphorylated tau 181 (p‐tau 181) in undiluted human plasma and serum. This work establishes a reagent‐free sensing strategy that eliminates external redox probes, offering a limit of detection of 980 fg mL^−1^ and sensitivity of 4.39 µA/(pg mL^−1^). The synergistic interplay between the redox‐active pPhR and conductive, nanostructured polypyrrole matrix amplifies signal transduction, enhances binding site accessibility, and improves structural stability under physiological conditions.

The platform further integrates a custom handheld potentiostat and a machine learning‐enabled web interface for automated classification and regression of electrochemical data, achieving 100% classification accuracy without operator bias. The sensor performs robustly across anticoagulant‐treated samples (EDTA, heparin), retains accuracy in complex biofluids, and significantly lowers cost compared to SIMOA or ELISA‐based assays.

This work lays the foundation for a scalable, AI‐integrated diagnostic platform that aligns with WHO's vision for equitable, decentralized diagnostics. Beyond Alzheimer's disease, the modular sensor architecture can be adapted for other neurodegenerative diseases—including Parkinson's and frontotemporal dementia—by targeting biomarkers such as α‐synuclein and p‐tau 217. Future developments will focus on multiplexed sensor arrays, mobile health integration, and clinical validation in diverse populations, accelerating translation toward real‐world, accessible neurodiagnostics.

## Conflict of Interest

The authors declare no conflict of interest.

## Supporting information



Supporting Information

## Data Availability

The data that support the findings of this study are available on request from the corresponding author. The data are not publicly available due to privacy or ethical restrictions.
