# Supplementary material for: Redox‐Active Polyphenol Red Molecularly Imprinted Polymers on Porous Gold Electrodes for Ultrasensitive, AI‐Assisted Detection of Alzheimer's Biomarkers in Undiluted Biofluids
Source: Adv Healthc Mater. 2025 Sep 4;14(32):e03155. doi: 10.1002/adhm.202503155 (PMC12716194; doi:10.1002/adhm.202503155)
Supplement: Supplementary file 1 — Supporting Information [file ADHM-14-0-s001.docx]

**Supporting information**

**Redox-Active Polyphenol Red Molecularly Imprinted Polymers on Porous Gold Electrodes for Ultrasensitive, AI -Assisted Detection of Alzheimer’s Biomarkers in Undiluted Biofluids**

Sudhaunsh Deshpande^1^, Arjun Ajith Mohan^1^, Guoyi Liu^1,2^, Kryztof Pawlak^3^, Sanjiv Sharma^1*^

*^1^David Price Evans Global Health and Infectious Diseases Group, Pharmacology & Therapeutics, Institute of Systems, Institute of Systems, Molecular and Integrative Biology, University of Liverpool, Crown Street, Liverpool L69 7BE, United Kingdom.*

*^2^Key Laboratory of Optoelectronic Technology & Systems (Chongqing University), Chongqing 400044, China*

*^3^Materials Innovation Factory, University of Liverpool, 51 Oxford Street, Liverpool L7 3NY*


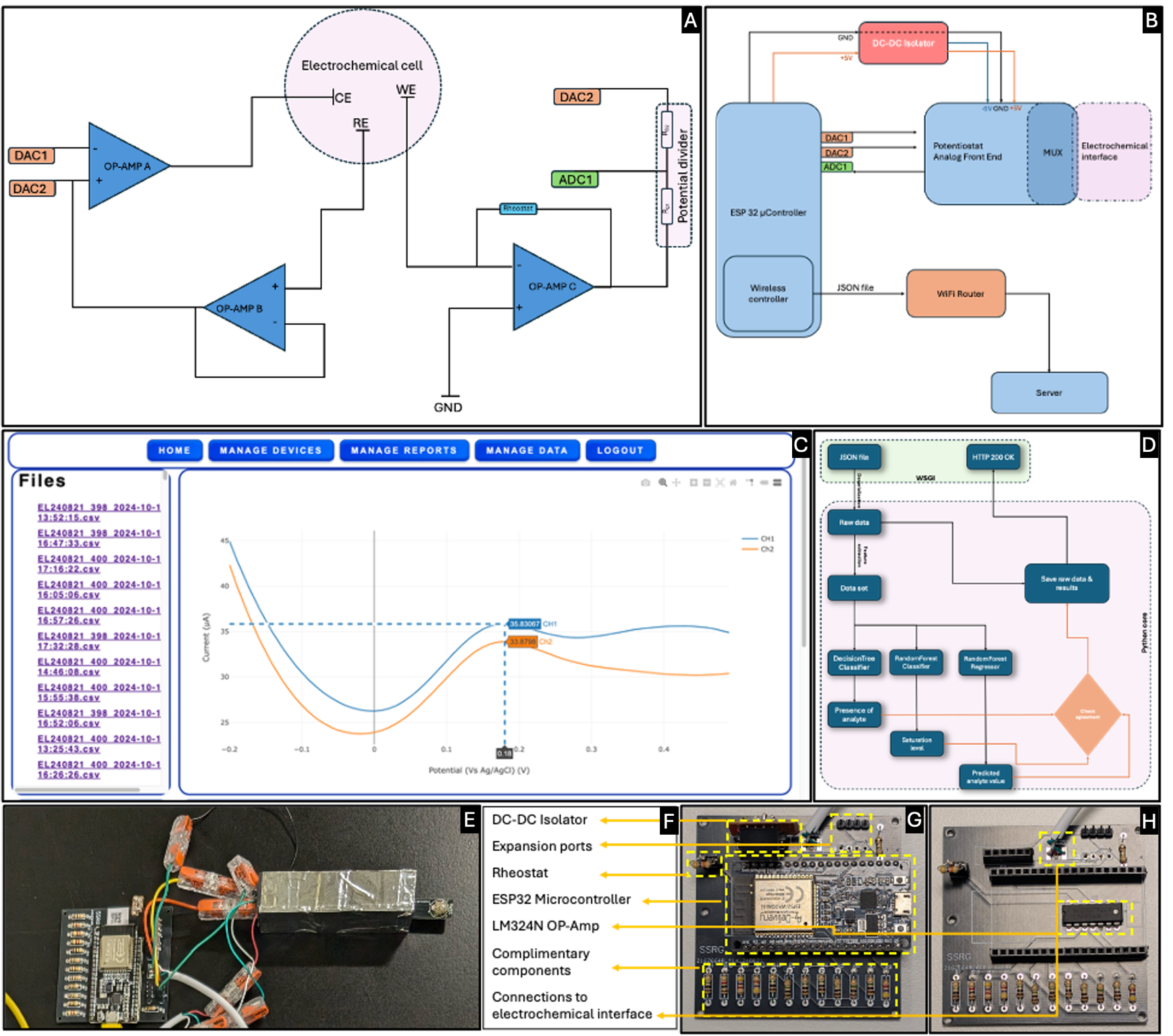


**Supporting Figure S1: Overview of the bioinstrumentation: A)** Circuit workflow of the potentiostat. **B)** Workflow chart showing the program function in the bioinstrumentation. **C)** Screenshot of the web app data view mode. **D)** Workflow chart of the machine learning algorithm. **E)** Image of the bioinstrumentation in use. **F)** List of components and parts. **G)** Image of the instrument. **H)** Image of the potentiostat motherboard without the microcontroller.

**
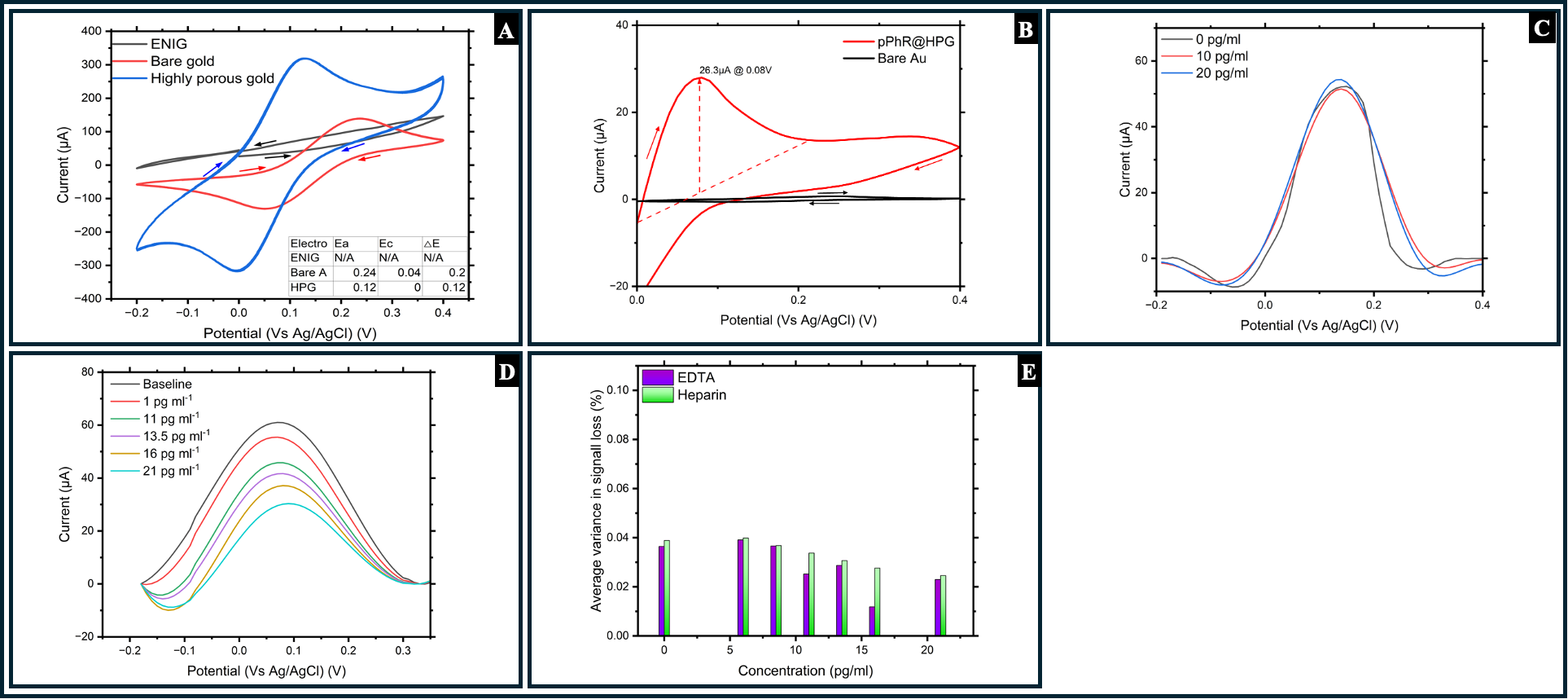
**

**Supporting Figure S2:** **Sensor Performance and optimisation: A)** Cyclic voltammograms of ENIG surface finish (**–**), Bare gold after surface modification (**–**), and highly porous gold (**–**) on PCB electrodes in a solution of 5mM Fe^2+^, Fe^3+^ ions and 100mM KCl as the supporting electrolyte. **B)** Cyclic voltammogram of Bare gold after PCB electrode (**–**) and poly-Phenol Red molecularly imprinted polymer (pPhR MIP) on highly porous gold (HPG) (**–**) in 10mM phosphate buffered saline (PBS). **C)** Differential Pulsed voltammograms of non-imprinted polymer (NIP) on HPG in spiked human K2EDTA plasma incubated for 30 minutes. **D)** Differential Pulsed voltammograms of MIP sensors in PBS incubated for 30 minutes. **E)** Bar graph depicting the signal loss variation in 20 samples in human plasma with K2EDTA and heparin as the anticoagulant.


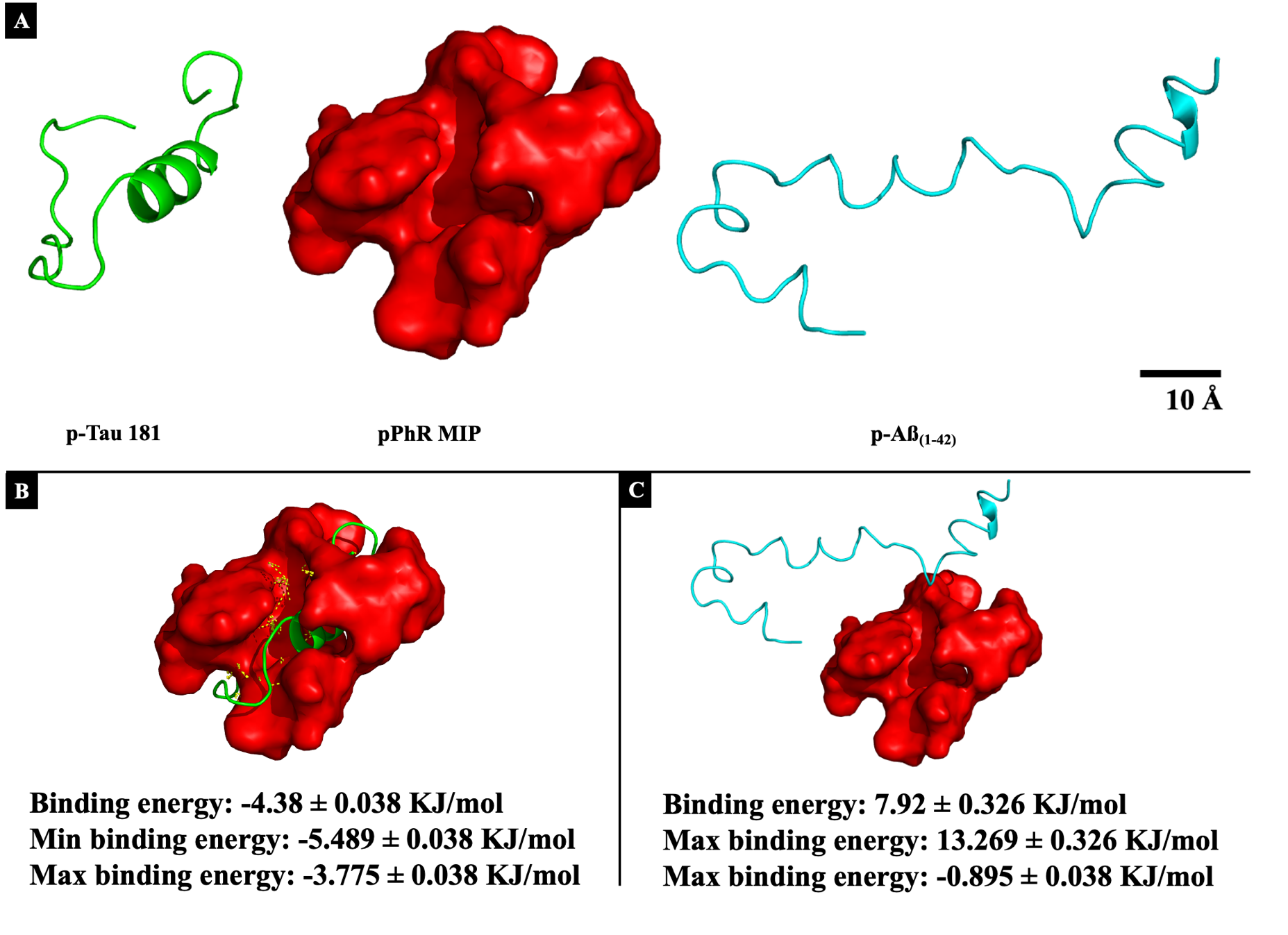


**Supporting Figure S3: Molecular modelling of the pPhR MIP's selective binding to p-tau 181: A)** Molecular representations of the target analyte, phosphorylated tau protein (p-tau 181), the poly-phenol red molecularly imprinted polymer (pPhR MIP), and the non-target control peptide, phospho-amyloid beta 1-42 (p-Aβ _(1-42)_). All structures are depicted at the same scale and were rendered using PyMOL. **B)** Results from a molecular docking simulation illustrating the favourable binding interaction between the pPhR MIP and its target, p-tau 181. The calculated binding energies and key interacting residues at the binding interface are shown. **C)** Corresponding simulation demonstrating the high selectivity of the pPhR MIP, as evidenced by the lack of significant binding interaction with the non-target p-Aβ _(1-42)_.

|  | SIMOA^1^ | ELISA^2^ | MIP (pPhR) |
| --- | --- | --- | --- |
| Limit of Detection | 724 fg ml^-1^ | 15.6 pg ml^-1^ | 980 fg ml^-1^ |
| Time required for assay | 60 minutes | 15 hours | 30 minutes |
| Cost per assay | US$ 677.20 | US$ 623.03 | US$ 0.68 |
| Equipment required | SIMOA analyser | 96 well plate reader | Potentiostat |
| Sample preparation | Required | Required | Not required |

**References:**

1. Quanterix. Simoa® pTau-181 Advantage V2.1 Kit HD-1/HD-X Data Sheet. Quanterix; 2022.
2. ThermoFisher Scientific. Human Tau (Phospho) [pT181] ELISA Kit. ThermoFisher 2018.

**Supporting Table 1**: Table showing the limit of Detection, time required for assay, and cost per assay, equipment requirement, and if sample preparation is required.
